# Engaging End Users in Designing Systems and Hardware for a Socially Assistive Robot

**DOI:** 10.1093/geroni/igaa057.3002

**Published:** 2020-12-16

**Authors:** Pamela Cacchione, Caio Mucchiani, Kristine Lima, Ross Mead, Mark Yim, Michelle Johnson

**Affiliations:** 1 University of Pennsylvania, Philadelphia, Pennsylvania, United States; 2 Semio AI, Venice, California, United States

## Abstract

Development of low-cost robots to assist older adults requires the input of end users: older adults, paid caregivers and clinicians. This study builds on prior work focused on the task investigation and deployment of mobile robots in a Program of All-inclusive Care for the Elderly. We identified hydration, walking and reaching as tasks appropriate for the robot and helpful to the older adults. In this study we investigated the design specifications for a socially assistive robot to perform the above tasks. Through focus groups of clinicians, older adults and paid caregivers we sought preferences on the design specifications. Using conventional content analysis, the following four themes emerged: the robot must be polite and personable; science fiction or alien like; depends on the need of the older adult; and multifaceted to meet the needs of older adults. These themes were used in the design and deployment of the Quori robot.

